# Comparative effectiveness of amlodipine versus nifedipine prescribed at discharge in patients with ischemic stroke or transient ischemic attack: A nationwide 5-year follow-up registry study

**DOI:** 10.1371/journal.pmed.1005277

**Published:** 2026-10-12

**Authors:** Xinya Li, Xue Xia, Meng Gao, Qin Xu, Yijun Zhang, Jing Jing, Yi Zhai, Xia Meng, Anxin Wang

**Affiliations:** 1 Department of Neurology, Beijing Tiantan Hospital, Capital Medical University, Beijing, China; 2 China National Clinical Research Center for Neurological Diseases, Beijing Tiantan Hospital, Capital Medical University, Beijing, China; 3 Department of Clinical Epidemiology and Clinical Trial, Capital Medical University, Beijing, China; 4 Department of Epidemiology, Beijing Neurosurgical Institute, Beijing Tiantan Hospital, Capital Medical University, Beijing, China; Columbia University, UNITED STATES OF AMERICA

## Abstract

**Background:**

Amlodipine and nifedipine are the most commonly prescribed calcium channel blockers (CCB) for patients suffering from ischemic stroke or transient ischemic attack (TIA). Nevertheless, there is a scarcity of direct comparative evidence on their effectiveness. This study aimed to compare the effectiveness of amlodipine and nifedipine in patients with ischemic stroke or TIA.

**Methods and findings:**

This observational study was based on the Third China National Stroke Registry, which recruited consecutive adult patients with ischemic stroke or TIA from 2015 to 2018. In this study, ischemic stroke or TIA patients were prescribed either amlodipine or nifedipine at discharge. The primary outcome was the distribution of modified Rankin Scale (mRS) score, which assesses functional outcomes in patients with stroke, at 5 years. The intergroup differences were examined using ordinal logistic regression, yielding odds ratios (OR) and 95% confidence intervals (CI). Patient age, gender, body mass index, systolic blood pressure at admission, and onset date were adjusted. To improve baseline characteristic balance, inverse probability of treatment weighting (IPTW) was implemented. A total of 2,859 eligible patients were included, and there were 1847 patients in the amlodipine group and 1,058 patients in the nifedipine group after IPTW. The amlodipine group demonstrated significantly lower 5-year mRS scores in comparison to the nifedipine group, with an OR of 0.76 (95% CI [0.65, 0.89]; *p* < 0.001). A total of 956 (51.76%) amlodipine users and 632 (59.74%) nifedipine users achieved a mRS score > 0 at 5 years, with an OR of 0.71 (95% CI [0.61, 0.83]; *p* < 0.001). The sensitivity analyses yielded consistent results. The main limitations include the inherent observational design, the exclusive reliance on registry data, and relatively high loss-to-follow-up rate at 5 years.

**Conclusions:**

This study revealed that amlodipine, compared to nifedipine, may be associated with improved functional recovery in ischemic stroke or TIA patients. Randomized controlled trials are warranted to confirm these findings and refine CCB therapy.

## Author summary

**Why was this study done?:**

- Amlodipine and nifedipine are the two most frequently prescribed calcium channel blockers (CCB) for ischemic stroke or transient ischemic attack (TIA) patients.
- There is an evidence gap on comparative effectiveness of amlodipine versus nifedipine for ischemic stroke or TIA patients.

**What did the researchers do and find?:**

- Leveraging the Third China National Stroke Registry, we compared 5-year functional outcomes between amlodipine and nifedipine users with balanced baseline characteristics.
- Amlodipine users showed better odds of favorable functional recovery versus nifedipine, with consistent results across sensitivity analyses.

**What do these findings mean?:**

- Amlodipine may be the preferred CCB for ischemic stroke or TIA patients seeking improved long-term functional outcomes.
- Limitations include the observational design and reliance on registry data, emphasizing the need for prospective randomized controlled trials to validate these results.

## Abstract 3

In a nationwide registry study, Xinya Li and colleagues compare the effectiveness of two calcium channel blockers prescribed at discharge of hospitalization due to stroke on patient functional outcomes.

## 1 Introduction

Globally, stroke ranks as the third leading cause of death and the fourth leading contributor to disability-adjusted life-years [1,2]. Among all new stroke cases, ischemic stroke constitutes the largest proportion, accounting for 65.3% of incidents [2]. Transient ischemic attack (TIA), often regarded as a critical warning sign for ischemic stroke, underscores the urgency of addressing this condition [3,4]. While considerable research advances have been achieved in acute-phase management, secondary prevention, and rehabilitation therapies, a notable proportion of patients continue to experience suboptimal functional recovery [5–7]. Given these challenges, the identification of more effective and safe therapeutic strategies in the long term has emerged as a pivotal direction to further enhance patient outcomes and reduce the disease burden, highlighting the need for continued innovation in stroke care [8].

Hypertension, as a well-established risk factor for stroke, has garnered significant attention regarding its therapeutic management [9]. Among antihypertensive agents, calcium channel blockers (CCB) hold a prominent position due to their pronounced ability to relax vascular smooth muscle, promote vasodilation, and reduce peripheral vascular resistance [10]. Amlodipine and nifedipine stand out as the two most commonly used CCB in clinical practice. However, there is currently a lack of comparative evidence for these two agents in patients with ischemic stroke or TIA, posing challenges to clinical drug selection and patient adherence. Considering the differences in molecular structure, dosage form design, and pharmacokinetics between them, we hypothesize that amlodipine may confer greater functional improvement compared to nifedipine in this patient population [11,12].

To validate our hypothesis, we conducted an observational study utilizing a nationwide registry database to compare the effectiveness of amlodipine versus nifedipine in patients with ischemic stroke or TIA, to furnish actionable evidence for optimizing clinical decision-making in cerebrovascular care.

## 2 Methods

### 2.1 Data source

The study’s source cohort was derived from the Third China National Stroke Registry (CNSR-III), initiated by the China National Clinical Research Center for Neurological Diseases. CNSR-III was a representative nationwide clinical registry specifically targeting ischemic stroke and TIA cases from 201 hospitals that cover 22 provinces in China [13]. From August 2015 to March 2018, this registry systematically included consecutive adult patients presenting with ischemic stroke or TIA who sought medical care within 7 days of symptom onset. The protocol of the CNSR-III study was approved by the ethics committee at Beijing Tiantan Hospital (IRB approval number: KY2015-001-01) and all participating centers. All participants enrolled in the CNSR-III provided written informed consent before enrollment. A total of 15,166 patients fulfilled inclusion criteria and submitted complete baseline data. Detailed methodological and demographic characteristics of this registry have been extensively documented in prior publications [13].

### 2.2 Study population

Eligible patients were those with a pre-stroke modified Rankin Scale (mRS) score of 0, diagnosis of ischemic stroke or TIA, hospital admission within 7 days after symptom onset, and prescribed either amlodipine or nifedipine monotherapy at discharge. The diagnosis of acute ischemic stroke followed the World Health Organization (WHO) criteria and was confirmed by magnetic resonance imaging (MRI) or brain computed tomography (CT) [14,15]. Individuals discharged with concurrent prescriptions for antihypertensive medications beyond the study drugs were systematically excluded. This study was reported as per the Reporting of Studies Conducted using Observational Routinely-Collected Data (RECORD) guideline (S1 Checklist).

### 2.3 Exposure

Patients were categorized into two groups according to their prescribed antihypertensive regimen at discharge: the amlodipine group and the nifedipine group. Amlodipine has an elimination half-life of ~30–50 hours. Immediate-release nifedipine exhibits a biphasic half-life of ~2.5–3 hours and 5 hours, respectively, whereas the controlled-release formulations have a half-life of ~2–3 hours. All medication-related data, including pharmacological classifications, treatment durations, and drug designations, were prospectively collected by research personnel with specialized training. Subsequently, these data underwent a comprehensive verification process, involving comparative analysis against authenticated medical directives or prescription documentation.

### 2.4 Outcomes and follow-up

The primary outcome was the distribution of mRS score at 5 years. Secondary outcomes included: (1) the distribution of mRS score at 1 year; (2) a mRS score > 0 at 5 years; (3) stroke recurrence in 5 years; and (4) all-cause mortality in 5 years. The mRS score of 0 signifies complete functional recovery and returned to pre-stroke condition, with no symptoms [16]. Conversely, a mRS score > 0 indicates persistent functional deficits or residual neurological impairments. The design of this study was presented in Fig 1.

**Fig 1:**
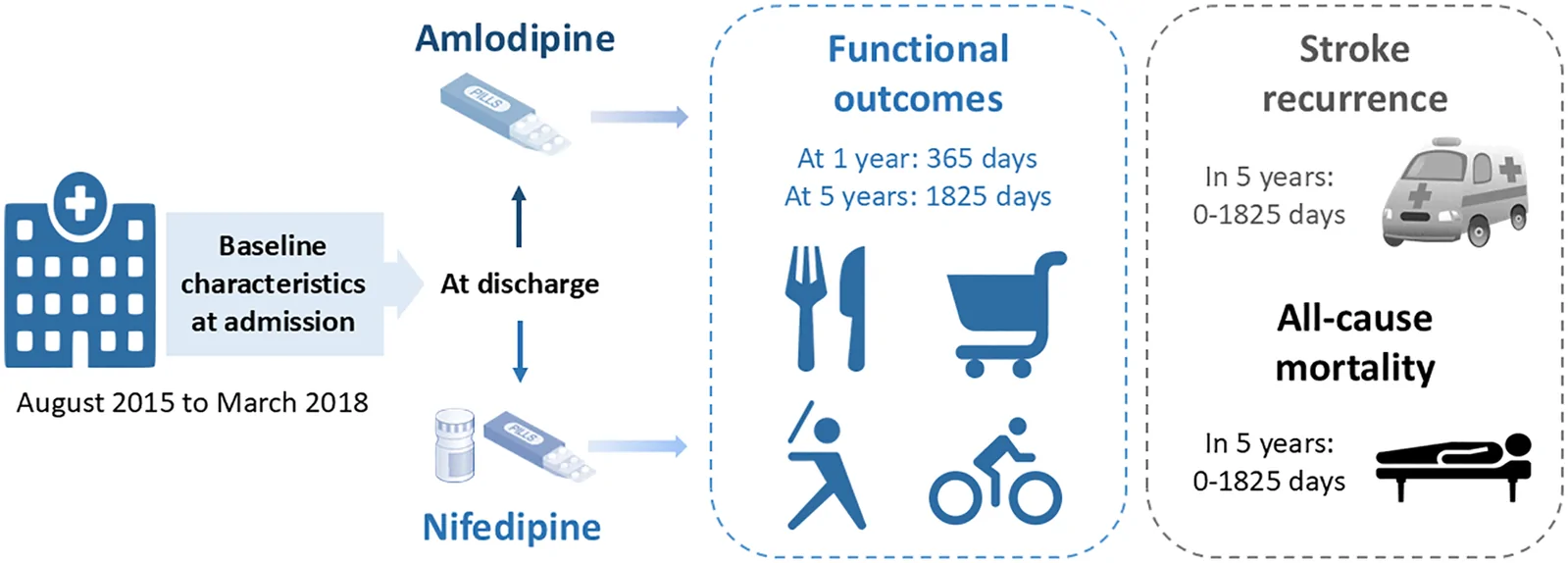
Study design. IPTW, inverse probability of treatment weighting.

The follow-up was conducted at 1 year and 5 years after enrollment. At each follow-up, proficient research coordinators reached out to participants via telephone, and the following data were collected: mRS score, stroke recurrence (including the date of onset when relevant), and mortality (including the date when applicable). In addition, adherence to the medication regimens at discharge was recorded at 1-year follow-up.

### 2.5 Baseline characteristics

The baseline demographic and clinical profiles included patient gender, age, body mass index (BMI), the onset date of the index event, admission diagnoses (ischemic stroke or TIA), neurological severity assessed by the total National Institutes of Health Stroke Scale (NIHSS) score upon admission, the systolic blood pressure (SBP), and diastolic blood pressure (DBP) at admission, and disease history (stroke or TIA, lipid metabolism disorders, diabetes, and heart diseases). Blood pressure was consistently recorded upon patient admission, incorporating bilateral arm measurements (left/right). The average SBP and DBP values were then calculated as the mean of these paired readings. Medical history was systematically gathered through standardized in-person interviews during enrollment, with data integrity ensured via cross-validation against comprehensive medical records.

### 2.6 Statistical analysis

To ensure analytical integrity, the last observation carried forward method was applied for missing mRS scores during follow-up assessments. No data imputation was performed for stroke recurrence, all-cause mortality, or cardiovascular mortality. Baseline characteristics were thoroughly documented without any missing data. Descriptive statistics were utilized to summarize patient characteristics: continuous variables were reported as mean with standard deviation (SD) for normal distributions or median with interquartile range (IQR) for skewed data, while categorical variables were summarized as counts (percentages). To enhance baseline characteristic balance, inverse probability of treatment weighting (IPTW) was applied. A multivariable logistic regression model, incorporating baseline covariates at admission (patient gender, age, BMI, the onset date of the index event, diagnoses, NIHSS score and SBP at admission, and disease history), estimated the probability of each patient receiving amlodipine or nifedipine at discharge. Subsequently, patient-specific stabilized weights were derived from these propensity scores to construct a weighted dataset, within which main analyses were performed. To assess whether the baseline characteristics were balanced both before and after IPTW, we used standardized differences between treatment groups (with < 0.1000 indicating negligible differences).

Regarding the outcomes assessed via mRS score, ordinal logistic regression was applied to assess shifts in mRS distribution, while binary logistic regression was utilized to examine proportions achieving mRS > 0, yielding odds ratios (OR) and 95% confidence intervals (CI). For stroke recurrence, and all-cause mortality, hazard ratios (HR) with 95% CI were estimated via Cox proportional hazards models. Assessment of Schoenfeld residuals demonstrated proportional hazards compliance in Cox models. For all outcomes, time zero was defined as the time of patient discharge from the hospital. The logistic and Cox regression models were adjusted for patient age, gender, BMI, SBP at admission, and onset date. To assess the robustness of our findings against potential unmeasured confounding factors, we computed *E*-values alongside their corresponding 95% lower and upper confidence intervals (LCI/UCI) [17].

To evaluate the robustness of our findings, we conducted a comprehensive suite of sensitivity analyses. First, we compared results before IPTW application to quantify the raw treatment effect without weighting adjustments. Second, we employed propensity score matching (PSM) to balance baseline characteristics between the two groups. Third, we examined patients who maintained adherence to the treatment regimen in 1 year, considering the impact of patient medication adherence on treatment outcomes. Patient adherence was defined as the sustained and continuous intake of nifedipine or amlodipine (according to their corresponding groups) over a 1-year period, with no use of other CCB medications. Fourth, we conducted analyses in unadjusted models, to minimize bias arising from covariate adjustment. Fifth, patients lost to 5-year follow-up were excluded from our analyses, accounting for the influence of missing follow-up data. Sixth, to address missing data while acknowledging LOCF limitations, we adopted multiple imputation for missing mRS data. The variables incorporated in the multiple imputation process included patient age, gender, BMI, SBP at admission, and onset date. Five imputations were performed, and the mean absolute value of the imputed estimates was calculated to ensure robustness of the final results. Seventh, we conducted the analyses focusing on participants without prior antihypertensive drug therapy to minimize confounding from pre-existing treatment histories. Eighth, considering the impact of nifedipine formulations on clinical outcomes, we excluded patients using immediate-release nifedipine tablets and compared the outcome differences between amlodipine users and controlled released nifedipine users. Ninth, we performed an analysis using Fine-Gray competing risk models with death treated as the competing event for stroke recurrence. Tenth, we compared outcomes between the amlodipine and nifedipine groups separately among patients with and without recurrent stroke to account for the potential influence of stroke recurrence on outcomes.

The analytical plan for this research was finalized in October 2025. In March 2026, we made two revisions based on peer review comments: first, we applied stabilized weights in the IPTW procedure; second, we added the second and eighth sensitivity analysis. In June 2026, we further supplemented the ninth and 10th sensitivity analysis following the peer review feedback. All statistical procedures were executed using SAS software, version 9.4 (SAS Institute), with two-tailed *P*-values < 0.05 defining statistical significance.

### 2.7 AI statement

This study did not use any artificial intelligence (AI) tools in the production of research data, execution of statistical analysis, drafting of manuscript sections, or generation of scientific conclusions.

## 3 Results

### 3.1 Study population

From the CNSR-Ⅲ cohort, 15,166 patients were initially screened. After excluding 442 patients with pre-stroke mRS scores > 0, 7,518 patients not prescribed antihypertensive medication at discharge, and 4,347 patients receiving other antihypertensive agents, a total of 2,859 patients (1,801 in the amlodipine group; 1,058 in the nifedipine group) were included in the study (Fig 2). Following IPTW, there were 1847 patients in the amlodipine group and 1,058 patients in the nifedipine group, and the baseline characteristics were well balanced across all variables except for BMI, which showed a standardized difference of 0.1305 (Table 1; Table A in S1 Appendix). Males constituted 66.49% of amlodipine users and 65.78% of nifedipine users, and the median age was 63 and 62, respectively. Regarding diagnosis, 93.44% and 93.38% of patients were ischemic stroke cases in the amlodipine and nifedipine groups, respectively. Upon admission, the median SBP was 153 (IQR: 140–167.5) mmHg in the amlodipine group and 155 (IQR: 140–170) mmHg in the nifedipine group. Moreover, the median DBP was 89 (IQR: 80–98) mmHg and 90 (IQR: 80–99) mmHg for amlodipine and nifedipine users, respectively. Amlodipine tablets were administered once daily. Regarding the formulation of nifedipine, 497 patients were administered controlled-release nifedipine (once daily), while the remaining participants received immediate-release nifedipine tablets (three times daily). During the 5-year follow-up, 464 patients were lost to follow-up, with 292 amlodipine users and 172 nifedipine users.

**Table 1:** Baseline characteristics after inverse probability of treatment weighting.

Characteristics	No. (%) or median (P25, P75)		Standardized difference	*P* value
	Amlodipine (*n* = 1,847)	Nifedipine (*n* = 1,058)		
Male	1,228 (66.49)	696 (65.78)	0.0148	0.7026
Age	63 (54, 70)	62 (54, 70)	0.0306	0.2676
BMI	24.22 (22.49, 26.29)	24.79 (22.85, 27.04)	0.1305	0.4464
Diagnosis			0.0026	0.9266
Ischemic stroke	1,726 (93.44)	988 (93.38)		
Transient ischemic attack	121 (6.56)	70 (6.62)		
NIHSS total score upon admission	3 (1, 5)	3 (1, 5)	0.0040	0.4266
SBP upon admission (mmHg)	153 (140, 167.5)	155 (140, 170)	0.0713	0.2450
DBP upon admission (mmHg)	89 (80, 98)	90 (80, 99)	0.0693	0.3443
Disease history				
Stroke or transient ischemic attack	339 (18.35)	224 (21.17)	0.0708	0.0567
Lipid metabolism disorders	201 (10.88)	95 (8.98)	0.0637	0.1237
Diabetes	404 (21.87)	251 (23.72)	0.0441	0.2474
Heart diseases	203 (10.99)	115 (10.87)	0.0039	0.9443

*P < 0.05; Between-group differences in binary data were compared using the χ^2^ test or Fisher’s exact test, and the Wilcoxon rank-sum test was applied for continuous variables. Abbreviations: BMI, Body mass index; DBP, Diastolic blood pressure; ICAS, Intracranial atherosclerotic stenosis; mmHg, Millimeter of mercury; NIHSS, National Institute of Health stroke scale; P25, 25th percentile; P75, 75th percentile; SBP, Systolic blood pressure.

**Fig 2:**
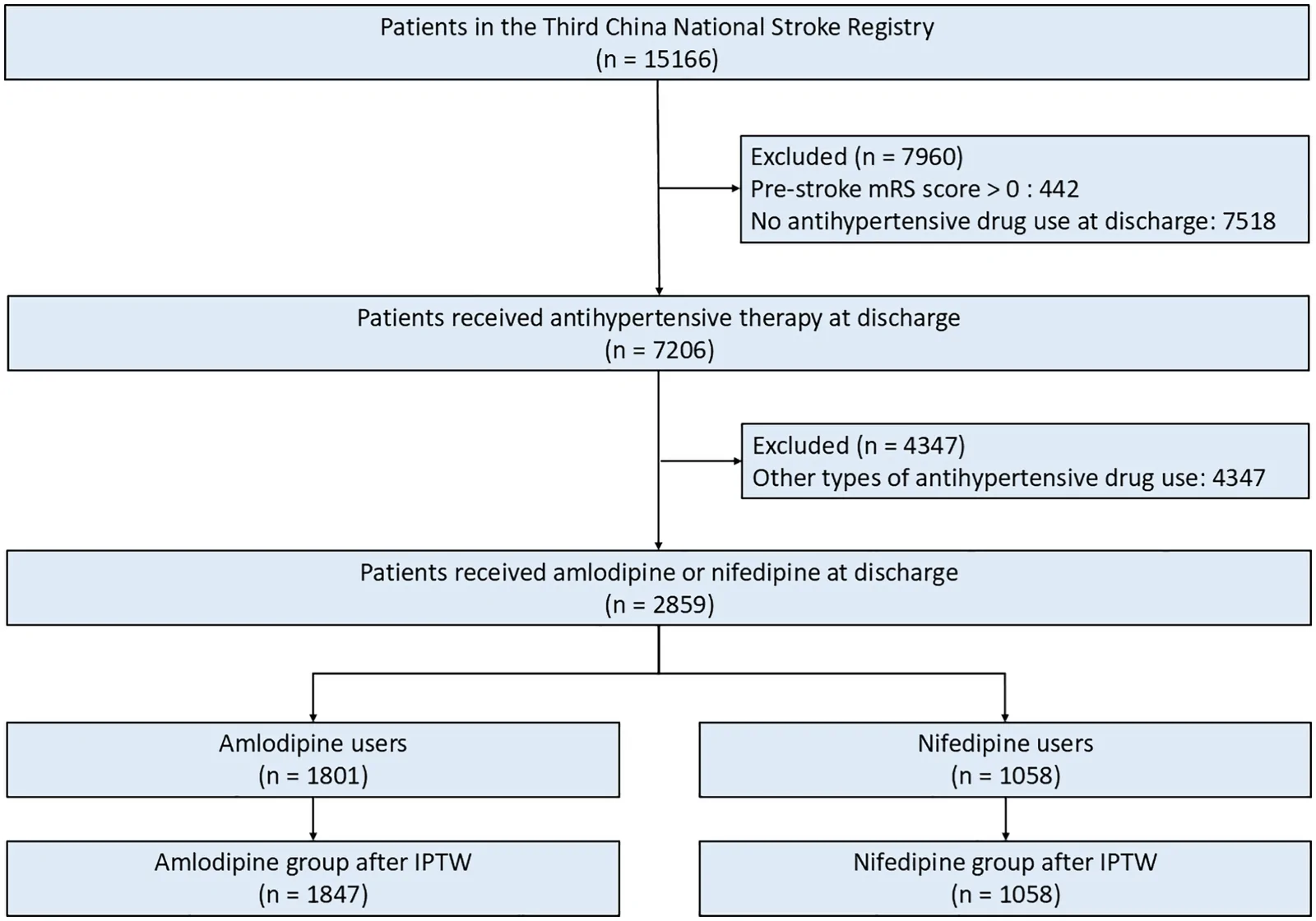
Flowchart of the study. IPTW, inverse probability of treatment weighting; mRS, modified Rankin Scale.

### 3.2 Primary outcomes

The amlodipine group exhibited significantly lower 5-year mRS scores compared to the nifedipine group, with an OR of 0.76 (95% CI [0.65, 0.89]; *p* < 0.001) and an *E*-value of 1.92 (95% UCI 1.50) (Table 2, Fig 3). Sensitivity analyses reported consistent findings. Specifically, in the sensitivity analyses prior to IPTW, utilizing PSM, involving patients who adhered to the treatment regimen within 1 year, using unadjusted models, excluding patients lost to follow-up at 5 years, addressing missing mRS data using multiple imputation, in those without a prior history of antihypertensive drug therapy, in amlodipine users and controlled released nifedipine users, using Fine–Gray competing risk models, and in patients without recurrent stroke, the ORs were 0.78 (95% CI [0.68, 0.90]; *p* < 0.001), 0.77 (95% CI [0.65, 0.90]; *p* = 0.001), 0.73 (95% CI [0.61, 0.86]; *p* < 0.001), 0.79 (95% CI [0.69, 0.91]; *p* < 0.001), 0.76 (95% CI [0.65, 0.89]; *p* < 0.001), 0.81 (95% CI [0.70, 0.93]; *p* = 0.003), 0.79 (95% CI [0.62, 0.99]; *p* = 0.044), 0.74 (95% CI [0.62, 0.90]; *p* = 0.002), 0.77 (95% CI [0.67, 0.89]; *p* < 0.001), and 0.74 (95% CI [0.63, 0.87]; *p* < 0.001), respectively (Table 3; Tables B–K in S1 Appendix).

**Table 2:** Primary and secondary outcomes of amlodipine users vs. nifedipine users.

Outcome measures	No of events (%)		OR/HR (95% CI)	*P* value	*E* value (95% UCI)
	Amlodipine (*n* = 1,847)	Nifedipine (*n* = 1,058)			
**Primary outcomes**					
mRS score at 5 years			0.77 (0.67, 0.89)	<0.001	1.92 (1.50)
0	890 (48.19)	426 (40.26)			
1	517 (27.99)	362 (34.22)			
2	163 (8.83)	100 (9.45)			
3	88 (4.76)	47 (4.44)			
4	41 (2.22)	29 (2.74)			
5	17 (0.92)	12 (1.13)			
6	131 (7.09)	82 (7.75)			
**Secondary outcomes**					
mRS score at 1 year			0.76 (0.66, 0.88)	<0.001	1.96 (1.53)
0	902 (48.84)	434 (41.02)			
1	630 (34.11)	418 (39.51)			
2	151 (8.18)	106 (10.02)			
3	75 (4.06)	52 (4.91)			
4	42 (2.27)	26 (2.46)			
5	10 (0.54)	6 (0.57)			
6	37 (2.00)	16 (1.51)			
mRS score > 0 at 5 years	956 (51.76)	632 (59.74)	0.71 (0.61, 0.83)	<0.001	2.17 (1.70)
Stroke recurrence in 5 years	249 (13.48)	147 (13.89)	0.98 (0.80, 1.20)	0.835	1.16 (1.00)
All-cause mortality in 5 years	131 (7.09)	82 (7.75)	0.89 (0.67, 1.17)	0.393	1.50 (1.00)

Patient age, gender, body mass index, systolic blood pressure at admission, and onset date were adjusted. Regarding the outcomes of mRS score, ordinal logistic regression was applied, while binary logistic regression was utilized to examine proportions achieving mRS > 0. For stroke recurrence and all-cause mortality, Cox proportional hazards models were used. Abbreviations: CI, confidence interval; HR, hazard ratio; mRS, modified Rankin Scale; NA, not applicable; OR, odds ratio; UCI, upper confidence interval.

**Table 3:** Primary and secondary outcomes of amlodipine users vs. nifedipine users before inverse probability of treatment weighting.

Outcome measures	No of events (%)		OR/HR (95% CI)	*P* value	*E* value (95% UCI)
	**Amlodipine (*n* = 1,801)**	**Nifedipine (*n* = 1,058)**			
**Primary outcomes**					
mRS score at 5 years			0.78 (0.68, 0.90)	<0.001	1.88 (1.46)
0	862 (47.86)	423 (39.98)			
1	505 (28.04)	363 (34.31)			
2	161 (8.94)	100 (9.45)			
3	87 (4.83)	48 (4.54)			
4	39 (2.17)	30 (2.84)			
5	18 (1.00)	12 (1.13)			
6	129 (7.16)	82 (7.75)			
**Secondary outcomes**					
mRS score at 1 year			0.76 (0.66, 0.88)	<0.001	1.96 (1.53)
0	875 (48.58)	430 (40.64)			
1	611 (33.93)	416 (39.32)			
2	152 (8.44)	107 (10.11)			
3	75 (4.16)	55 (5.20)			
4	42 (2.33)	27 (2.55)			
5	10 (0.56)	6 (0.57)			
6	36 (2.00)	17 (1.61)			
mRS score > 0 at 5 years	939 (52.14)	635 (60.02)	0.71 (0.61, 0.84)	<0.001	2.17 (1.67)
Stroke recurrence in 5 years	248 (13.77)	153 (14.46)	0.96 (0.79, 1.18)	0.698	1.25 (1.00)
All-cause mortality in 5 years	129 (7.16)	82 (7.75)	0.92 (0.69, 1.21)	0.531	1.39 (1.00)

Patient age, gender, body mass index, systolic blood pressure at admission, and onset date were adjusted. Regarding the outcomes of mRS score, ordinal logistic regression was applied, while binary logistic regression was utilized to examine proportions achieving mRS > 0. For stroke recurrence and all-cause mortality, Cox proportional hazards models were used. Abbreviations: CI, confidence interval; HR, hazard ratio; mRS, modified Rankin Scale; NA, not applicable; OR, odds ratio; UCI, upper confidence interval.

**Fig 3:**
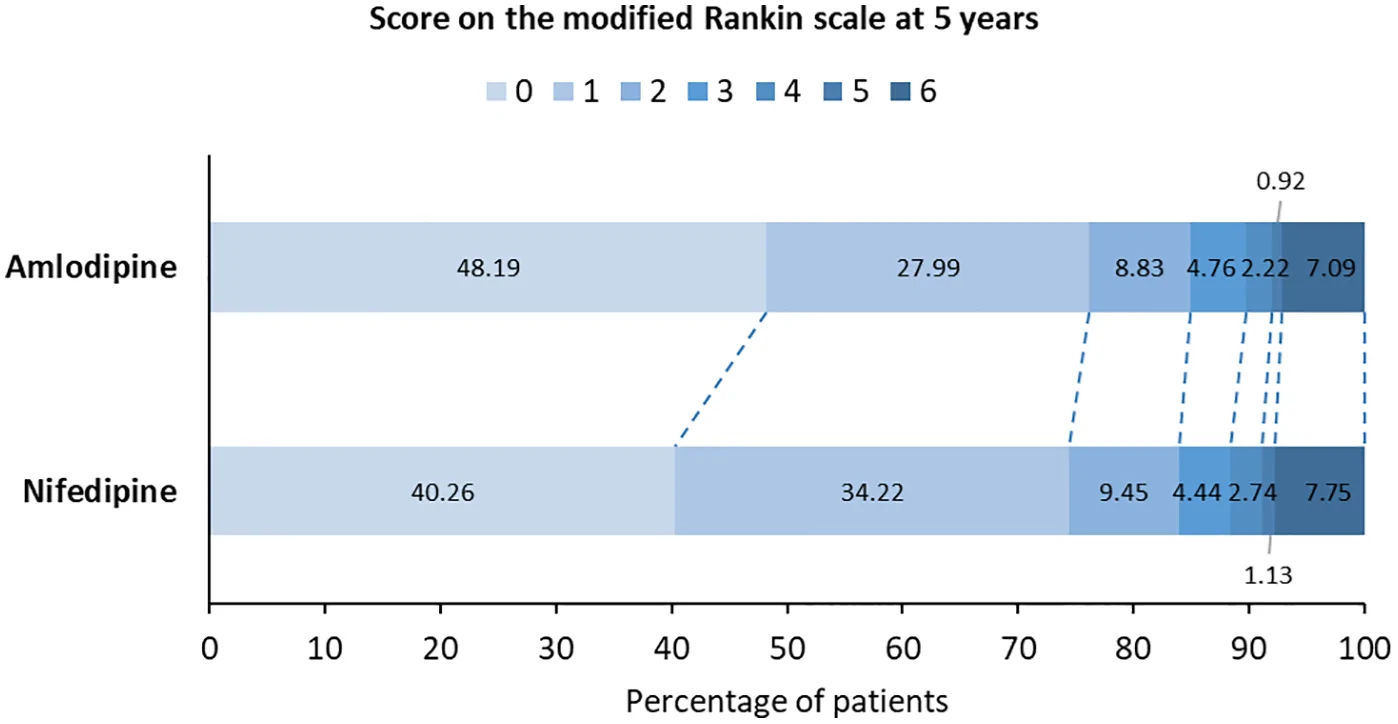
Distribution of scores on the modified Rankin scale of amlodipine users and nifedipine users at 5 years.

### 3.3 Secondary outcomes

At the 1-year follow-up, amlodipine users exhibited significantly lower mRS scores compared to nifedipine users, with an OR of 0.76 (95% CI [0.66, 0.88]; *p* < 0.001) and an *E*-value of 1.96 (95% UCI 1.53) (Table 2). A total of 956 (51.76%) amlodipine users and 632 (59.74%) nifedipine users achieved a mRS score > 0 at 5 years, with an OR of 0.71 (95% CI [0.61, 0.83]; *p* < 0.001) and an *E*-value of 2.17 (95% UCI 1.70) (Table 2, Fig 3). As for the mRS score at 1 year and mRS score > 0 at 5 years, all sensitivity analyses reported similar results. Regarding stroke recurrence and all-cause mortality in 5 years, no statistically significant difference was observed between the amlodipine and nifedipine groups (Tables 2 and 3; Tables B–K in S1 Appendix). For recurrent stroke, the amlodipine group had 229 cases (12.40%) of ischemic stroke and 20 cases (1.08%) of hemorrhagic stroke, whereas the nifedipine group had 130 cases (12.29%) of ischemic stroke and 17 cases (1.61%) of hemorrhagic stroke.

## 4 Discussion

This nationwide registry-based observational study conducted a comparative analysis of the patient outcome profiles of amlodipine and nifedipine in patients diagnosed with ischemic stroke or TIA. The findings indicated that, in contrast to nifedipine, the use of amlodipine may be associated with improved functional outcomes at both the 1-year and 5-year marks. When it came to stroke recurrence and all-cause mortality, there were no statistically significant disparities observed between the two treatment groups.

Our study indicated that amlodipine, compared to nifedipine, was associated with superior functional outcomes in patients with ischemic stroke or TIA. This difference can be attributed to several key mechanism hypotheses. First, amlodipine, as a third-generation long-acting CCB, exhibits a half-life of 30–50 hours, enabling beneficial effects on reducing systolic blood pressure variability-, as evidenced by a high trough-to-peak ratio [18,19]. In contrast, while nifedipine controlled-release formulations extend its duration of action, they still demonstrate greater blood pressure fluctuations compared to amlodipine [20,21]. Elevated blood pressure variability has been identified as an independent predictor of stroke recurrence, and amlodipine’s ability to mitigate abrupt blood pressure changes reduces secondary injury to neurons in the ischemic penumbra, thereby enhancing neurologic recovery [22,23].

Second, amlodipine also exerts anti-atherosclerotic effects by inhibiting calcium overload, enhancing endothelial function via nitric oxide release, and stabilizing plaques through reduced inflammation. The Prospective Randomized Evaluation of the Vascular Effects of Norvasc Trial (PREVENT) showed amlodipine had a significant effect in slowing the progression of carotid artery atherosclerosis [24]. Third, amlodipine offers synergistic management of comorbidities prevalent in stroke survivors, such as diabetes mellitus, coronary artery disease, and chronic kidney disease [25]. Unlike nifedipine, which may elevate uric acid levels and increase gout risk, amlodipine does not adversely affect glucose metabolism, improves renal perfusion, and reduces proteinuria, thereby delaying estimated glomerular filtration rate decline [11]. Finally, amlodipine appears to offer distinct advantages in improving outcomes for patients with neurological disorders. A registry study indicated that in Alzheimer’s disease or dementia with Lewy bodies/Parkinson’s disease dementia, mortality risk was significantly lower among amlodipine users versus other CCB. Additionally, Alzheimer’s patients on amlodipine showed a notably reduced stroke risk compared to those using alternative CCB [26]. In summary, amlodipine’s mechanisms, including stable blood pressure reduction, anti-atherosclerotic actions, and comorbidity management, collectively explain its superior functional prognosis in ischemic stroke or TIA patients, compared to nifedipine, making it the preferred choice for high-risk populations with multiple chronic conditions [27,28]. Notably, while we discussed potential mechanisms based on existing evidence, relevant biomarker validation remains absent. Future research could further investigate these underlying mechanisms and provide empirical validation.

In our study, several strengths presented themselves. First, based on a national registry, the data exhibit strong representativeness, providing an accurate reflection of the therapeutic situation for patients afflicted with ischemic stroke and TIA across China. Second, the research question holds substantial practical importance, as it offers valuable guiding perspectives for clinical practice by conducting a straightforward comparison of the treatment results between amlodipine and nifedipine. Third, following the application of IPTW, the baseline characteristics of the amlodipine and nifedipine groups were evenly matched, effectively reducing confounding bias. Fourth, we carried out comprehensive sensitivity analyses to guarantee the consistency and reliability of the study’s findings.

Nevertheless, this study comes with several limitations that warrant careful attention. First, due to the observational design of our research, our analysis merely uncovered a potential association between drug therapy and patient outcomes, making it difficult to confirm causality. Although our sensitivity analyses rigorously assessed the stability and credibility of our findings under various conditions and assumptions, the risk of unmeasured confounding factors, particularly factors such as drug doses and blood pressure changes during follow-up, cannot be overlooked. However, we utilized the *E*-values to gauge the resilience of our results against unmeasured confounders. Using the primary outcome from our main analysis as an illustrative example, an *E*-value of 1.92 (95% UCI: 1.50) quantified the minimum strength of association that an unmeasured confounder must demonstrate with both amlodipine use (compared to nifedipine) and lower mRS scores to fully explain the observed treatment-outcome association in our study. The *E*-value suggested that our findings exhibit substantial robustness against unmeasured confounding, as the required confounder magnitude exceeds available effect sizes in current evidence, thereby reinforcing the validity of our conclusions. In essence, future randomized controlled trials (RCT) are essential to corroborate our findings. Second, constrained by usage rates, our research questions were focused on the two most frequently prescribed CCB, thereby excluding other medications and inevitably limiting the scope of our conclusions. Third, the scarcity of data on adverse drug reactions related to amlodipine and nifedipine hindered our ability to compare outcomes in this regard. Fourth, the study analyzed 5-year mRS score as an ordinal outcome, which may violate the proportionality assumption. However, a binary mRS score was used as a secondary outcome to validate our findings. Fifth, the loss-to-follow-up rate in this study was relatively high, which may be attributable to factors such as the extended follow-up duration. Sixth, although there were no significant between-group differences in diagnosis, NIHSS total score, SBP, or DBP upon admission, the nifedipine group had marginally higher SBP and DBP at baseline, which may partially account for the more favorable outcomes observed in the amlodipine group. Seventh, only the mRS score-related outcomes reached statistical significance, whereas stroke recurrence and mortality, despite showing a consistent directional trend, did not achieve statistical significance. Therefore, the findings should be interpreted with caution. Finally, as this study was conducted within the Chinese population, caution must be exercised when extrapolating these findings to other nationalities and ethnic groups. Despite these constraints, the overall trends in our study results are still discernible. Looking ahead, further research is imperative to furnish additional evidence and foster a more holistic understanding on the CCB treatment in stroke care.

In conclusion, the observational research, which drew upon real-world data, revealed a possible advantage of amlodipine in terms of 5-year functional recovery in patients who had experienced ischemic stroke or TIA. When it came to stroke recurrence and all-cause mortality, there were no significant differences observed between the two medications. To solidify these observations and enhance the precision of CCB therapy for patients with ischemic stroke or TIA, further RCT are warranted.

## Supporting information

S1 Checklist S1 RECORD checklist: supporting information. (DOCX)

S1 Appendix Appendix Tables A–K. (DOCX)

## Abbreviations

AI: artificial intelligence
BMI: body mass index
CCB: calcium channel blockers
CI: confidence intervals
CNSR-III: Third China National Stroke Registry
CT: computed tomography
DBP: diastolic blood pressure
HR: hazard ratios
HR: hazard ratios
IPTW: inverse probability of treatment weighting
IQR: with interquartile range
MRI: magnetic resonance imaging
mRS: modified Rankin Scale
NIHSS: National Institutes of Health Stroke Scale
OR: odds ratios
PREVENT: Prospective Randomized Evaluation of the Vascular Effects of Norvasc Trial
PSM: propensity score matching
RCT: randomized controlled trials
RECORD: Reporting of Studies Conducted using Observational Routinely-Collected Data
SBP: systolic blood pressure
SD: standard deviation
TIA: transient ischemic attack
WHO: World Health Organization.
