# Supplementary material for: Comparative effectiveness of amlodipine versus nifedipine prescribed at discharge in patients with ischemic stroke or transient ischemic attack: A nationwide 5-year follow-up registry study
Source: PLoS Med. 2026 Oct 12;23(10):e1005277. doi: 10.1371/journal.pmed.1005277 (PMC13671639; doi:10.1371/journal.pmed.1005277)
Supplement: Supplementary file 2 [file pmed.1005277.s002.docx]

**Appendix**

Table A. Baseline characteristics before inverse probability of treatment weighting

Table B. Primary and secondary outcomes of amlodipine users and nifedipine users after propensity score matching

Table C. Primary and secondary outcomes of amlodipine users and nifedipine users who adhered to the treatment regimen in 1 year

Table D. Primary and secondary outcomes of amlodipine users and nifedipine users in unadjusted models

Table E. Primary and secondary outcomes of amlodipine users and nifedipine users with patients lost to follow-up at 5 years excluded

Table F. Primary and secondary outcomes of amlodipine users and nifedipine users with multiple imputation employed for missing mRS data

Table G. Primary and secondary outcomes of amlodipine users and nifedipine users who had no prior history of antihypertensive drug therapy

Table H. Primary and secondary outcomes of amlodipine users and controlled released nifedipine users

Table I. Primary and secondary outcomes of amlodipine users and nifedipine users using Fine-Gray competing risk models

Table J. Primary and secondary outcomes of amlodipine users and nifedipine users in patients with recurrent stroke

Table K. Primary and secondary outcomes of amlodipine users and nifedipine users in patients without recurrent stroke

**Table A. Baseline characteristics before inverse probability of treatment weighting**

| **Characteristics** | **No. (%) or median (P25, P75)** | | **Standardized difference** | **P value** |
| --- | --- | --- | --- | --- |
|  | **Amlodipine (n = 1801)** | **Nifedipine (n = 1058)** |  |  |
| Male | 1191 (66.13) | 694 (65.60) | 0.0113 | 0.7710 |
| Age | 63 (54, 69) | 62 (54, 70) | 0.0214 | 0.4456 |
| BMI | 24.49 (22.68, 26.67) | 25.01 (22.92, 27.34) | 0.1308 | 0.0003 |
| Diagnosis |  |  | 0.0012 | 0.9749 |
| Ischemic stroke | 1683 (93.45) | 989 (93.48) |  |  |
| Transient ischemic attack | 118 (6.55) | 69 (6.52) |  |  |
| NIHSS total score upon admission | 3 (1, 5) | 3 (1, 5) | 0.0592 | 0.5086 |
| SBP upon admission (mmHg) | 154 (140.5, 168) | 155 (140.5, 171) | 0.0862 | 0.0843 |
| DBP upon admission (mmHg) | 89.5 (80, 99) | 90 (80, 100) | 0.0631 | 0.2039 |
| Disease history |  |  |  |  |
| Stroke or transient ischemic attack | 362 (20.10) | 246 (23.25) | 0.0765 | 0.0468 |
| Lipid metabolism disorders | 173 (9.61) | 84 (7.94) | 0.0589 | 0.1326 |
| Diabetes | 413 (22.93) | 260 (24.57) | 0.0386 | 0.3174 |
| Heart diseases | 196 (10.88) | 113 (10.68) | 0.0065 | 0.8664 |

*P<0.05; Between-group differences in binary data were compared using the χ² test or Fisher’s exact test, and the Wilcoxon rank-sum test was applied for continuous variables. BMI, Body mass index; DBP, Diastolic blood pressure; ICAS, Intracranial atherosclerotic stenosis; mmHg, Millimeter of mercury; NIHSS, National Institute of Health stroke scale; P25, 25th percentile; P75, 75th percentile; SBP, Systolic blood pressure

**Table B. Primary and secondary outcomes of amlodipine users and nifedipine users after propensity score matching**

| **Outcome measures** | **Amlodipine (n = 1052)** | **Nifedipine (n = 1052)** |
| --- | --- | --- |
| **Primary outcomes** | | |
| **mRS score at 5 years** | | |
| Odds ratio (95% CI) | 0.77 (0.65, 0.90) | Reference |
| P value | 0.001 | Reference |
| E value (95% UCI) | 1.92 (1.46) | Reference |
| **Secondary outcomes** | | |
| **mRS score at 1 year** | | |
| Odds ratio (95% CI) | 0.79 (0.67, 0.93) | Reference |
| P value | 0.004 | Reference |
| E value (95% UCI) | 1.85 (1.36) | Reference |
| **mRS score > 0 at 5 years** |  |  |
| No of events (%) | 540 (51.33) | 632 (60.08) |
| Odds ratio (95% CI) | 0.70 (0.59, 0.83) | Reference |
| P value | <0.001 | Reference |
| E value (95% UCI) | 2.21 (1.70) | Reference |
| **Stroke recurrence in 5 years** | | |
| No of events (%) | 156 (14.83) | 152 (14.45) |
| Hazard ratio (95% CI) | 1.03 (0.83, 1.29) | Reference |
| P value | 0.788 | Reference |
| E value (95% LCI) | 1.21 (1.00) | Reference |
| **All-cause mortality in 5 years** | | |
| No of events (%) | 74 (7.03) | 81 (7.70) |
| Hazard ratio (95% CI) | 0.93 (0.67, 1.27) | Reference |
| P value | 0.627 | Reference |
| E value (95% UCI) | 1.36 (1.00) | Reference |

Patient age, gender, body mass index, systolic blood pressure at admission, and onset date were adjusted. Regarding the outcomes of mRS score, ordinal logistic regression was applied, while binary logistic regression was utilized to examine proportions achieving mRS > 0. For stroke recurrence and all-cause mortality, Cox proportional hazards models were used. CI, confidence interval; LCI, lower confidence interval; mRS, modified Rankin Scale; UCI, upper confidence interval

**Table C. Primary and secondary outcomes of amlodipine users and nifedipine users** **who adhered to the treatment regimen in 1 year**

| **Outcome measures** | **Amlodipine (n = 1228)** | **Nifedipine (n = 765)** |
| --- | --- | --- |
| **Primary outcomes** | | |
| **mRS score at 5 years** | | |
| Odds ratio (95% CI) | 0.73 (0.61, 0.86) | Reference |
| P value | <0.001 | Reference |
| E value (95% UCI) | 2.08 (1.60) | Reference |
| **Secondary outcomes** | | |
| **mRS score at 1 year** | | |
| Odds ratio (95% CI) | 0.70 (0.59, 0.83) | Reference |
| P value | <0.001 | Reference |
| E value (95% UCI) | 2.21 (1.70) | Reference |
| **mRS score > 0 at 5 years** |  |  |
| No of events (%) | 606 (49.35) | 453 (59.22) |
| Odds ratio (95% CI) | 0.67 (0.56, 0.81) | Reference |
| P value | <0.001 | Reference |
| E value (95% UCI) | 2.35 (1.77) | Reference |
| **Stroke recurrence in 5 years** | | |
| No of events (%) | 149 (12.13) | 96 (12.55) |
| Hazard ratio (95% CI) | 1.00 (0.77, 1.29) | Reference |
| P value | 0.968 | Reference |
| E value (95% LCI) | 1.00 (1.00) | Reference |
| **All-cause mortality in 5 years** | | |
| No of events (%) | 65 (5.29) | 49 (6.41) |
| Hazard ratio (95% CI) | 0.85 (0.59, 1.24) | Reference |
| P value | 0.405 | Reference |
| E value (95% UCI) | 1.63 (1.00) | Reference |

Patient age, gender, body mass index, systolic blood pressure at admission, and onset date were adjusted. Regarding the outcomes of mRS score, ordinal logistic regression was applied, while binary logistic regression was utilized to examine proportions achieving mRS > 0. For stroke recurrence and all-cause mortality, Cox proportional hazards models were used. CI, confidence interval; LCI, lower confidence interval; mRS, modified Rankin Scale; UCI, upper confidence interval

**Table D. Primary and secondary outcomes of amlodipine users and nifedipine users in unadjusted models**

| **Outcome measures** | **Amlodipine (n = 1847)** | **Nifedipine (n = 1058)** |
| --- | --- | --- |
| **Primary outcomes** | | |
| **mRS score at 5 years** | | |
| Odds ratio (95% CI) | 0.79 (0.69, 0.91) | Reference |
| P value | <0.001 | Reference |
| E value (95% UCI) | 1.85 (1.43) | Reference |
| **Secondary outcomes** | | |
| **mRS score at 1 year** | | |
| Odds ratio (95% CI) | 0.77 (0.67, 0.89) | Reference |
| P value | <0.001 | Reference |
| E value (95% UCI) | 1.92 (1.50) | Reference |
| **mRS score > 0 at 5 years** |  |  |
| No of events (%) | 956 (51.76) | 632 (59.74) |
| Odds ratio (95% CI) | 0.72 (0.62, 0.84) | Reference |
| P value | <0.001 | Reference |
| E value (95% UCI) | 2.12 (1.67) | Reference |
| **Stroke recurrence in 5 years** | | |
| No of events (%) | 249 (13.48) | 147 (13.89) |
| Hazard ratio (95% CI) | 0.97 (0.79, 1.18) | Reference |
| P value | 0.734 | Reference |
| E value (95% LCI) | 1.21 (1.00) | Reference |
| **All-cause mortality in 5 years** | | |
| No of events (%) | 131 (7.09) | 82 (7.75) |
| Hazard ratio (95% CI) | 0.91 (0.69, 1.20) | Reference |
| P value | 0.514 | Reference |
| E value (95% UCI) | 1.43 (1.00) | Reference |

Regarding the outcomes of mRS score, ordinal logistic regression was applied, while binary logistic regression was utilized to examine proportions achieving mRS > 0. For stroke recurrence and all-cause mortality, Cox proportional hazards models were used. CI, confidence interval; LCI, lower confidence interval; mRS, modified Rankin Scale; UCI, upper confidence interval

**Table E. Primary and secondary outcomes of amlodipine users and nifedipine users** **with patients lost to follow-up at 5 years excluded**

| **Outcome measures** | **Amlodipine (n = 1555)** | **Nifedipine (n = 886)** |
| --- | --- | --- |
| **Primary outcomes** | | |
| **mRS score at 5 years** | | |
| Odds ratio (95% CI) | 0.76 (0.65, 0.89) | Reference |
| P value | <0.001 | Reference |
| E value (95% UCI) | 1.96 (1.50) | Reference |
| **Secondary outcomes** | | |
| **mRS score at 1 year** | | |
| Odds ratio (95% CI) | 0.72 (0.62, 0.84) | Reference |
| P value | <0.001 | Reference |
| E value (95% UCI) | 2.12 (1.67) | Reference |
| **mRS score > 0 at 5 years** |  |  |
| No of events (%) | 746 (47.97) | 498 (56.21) |
| Odds ratio (95% CI) | 0.71 (0.60, 0.84) | Reference |
| P value | <0.001 | Reference |
| E value (95% UCI) | 2.17 (1.67) | Reference |
| **Stroke recurrence in 5 years** | | |
| No of events (%) | 196 (12.60) | 114 (12.87) |
| Hazard ratio (95% CI) | 0.98 (0.78, 1.24) | Reference |
| P value | 0.878 | Reference |
| E value (95% LCI) | 1.16 (1.00) | Reference |
| **All-cause mortality in 5 years** | | |
| No of events (%) | 21 (1.35) | 15 (1.69) |
| Hazard ratio (95% CI) | 0.84 (0.42, 1.64) | Reference |
| P value | 0.601 | Reference |
| E value (95% UCI) | 1.67 (1.00) | Reference |

Patient age, gender, body mass index, systolic blood pressure at admission, and onset date were adjusted. Regarding the outcomes of mRS score, ordinal logistic regression was applied, while binary logistic regression was utilized to examine proportions achieving mRS > 0. For stroke recurrence and all-cause mortality, Cox proportional hazards models were used. CI, confidence interval; LCI, lower confidence interval; mRS, modified Rankin Scale; UCI, upper confidence interval

**Table F. Primary and secondary outcomes of amlodipine users and nifedipine users with multiple imputation employed for missing mRS data**

| **Outcome measures** | **Amlodipine (n = 1847)** | **Nifedipine (n = 1058)** |
| --- | --- | --- |
| **Primary outcomes** | | |
| **mRS score at 5 years** | | |
| Odds ratio (95% CI) | 0.81 (0.70, 0.93) | Reference |
| P value | 0.003 | Reference |
| E value (95% UCI) | 1.77 (1.36) | Reference |
| **Secondary outcomes** | | |
| **mRS score at 1 year** | | |
| Odds ratio (95% CI) | 0.75 (0.65, 0.87) | Reference |
| P value | 0.0001 | Reference |
| E value (95% UCI) | 2.00 (1.56) | Reference |
| **mRS score > 0 at 5 years** |  |  |
| No of events (%) | 973 (52.68) | 625 (59.07) |
| Odds ratio (95% CI) | 0.75 (0.65, 0.88) | Reference |
| P value | 0.0004 | Reference |
| E value (95% UCI) | 2.00 (1.53) | Reference |
| **Stroke recurrence in 5 years** | | |
| No of events (%) | 249 (13.48) | 147 (13.89) |
| Hazard ratio (95% CI) | 0.98 (0.80, 1.20) | Reference |
| P value | 0.8349 | Reference |
| E value (95% LCI) | 1.16 (1.00) | Reference |
| **All-cause mortality in 5 years** | | |
| No of events (%) | 131 (7.09) | 82 (7.75) |
| Hazard ratio (95% CI) | 0.89 (0.67, 1.17) | Reference |
| P value | 0.3930 | Reference |
| E value (95% UCI) | 1.50 (1.00) | Reference |

Patient age, gender, body mass index, systolic blood pressure at admission, and onset date were adjusted. Regarding the outcomes of mRS score, ordinal logistic regression was applied, while binary logistic regression was utilized to examine proportions achieving mRS > 0. For stroke recurrence and all-cause mortality, Cox proportional hazards models were used. CI, confidence interval; LCI, lower confidence interval; mRS, modified Rankin Scale; UCI, upper confidence interval

**Table G. Primary and secondary outcomes of amlodipine users and nifedipine users who had no prior history of antihypertensive drug therapy**

| **Outcome measures** | **Amlodipine (n = 789)** | **Nifedipine (n = 368)** |
| --- | --- | --- |
| **Primary outcomes** | | |
| **mRS score at 5 years** | | |
| Odds ratio (95% CI) | 0.79 (0.62, 0.99) | Reference |
| P value | 0.044 | Reference |
| E value (95% UCI) | 1.85 (1.11) | Reference |
| **Secondary outcomes** | | |
| **mRS score at 1 year** | | |
| Odds ratio (95% CI) | 0.68 (0.54, 0.86) | Reference |
| P value | 0.001 | Reference |
| E value (95% UCI) | 2.30 (1.60) | Reference |
| **mRS score > 0 at 5 years** |  |  |
| No of events (%) | 391 (49.56) | 205 (55.71) |
| Odds ratio (95% CI) | 0.76 (0.59, 0.98) | Reference |
| P value | 0.034 | Reference |
| E value (95% UCI) | 1.96 (1.16) | Reference |
| **Stroke recurrence in 5 years** | | |
| No of events (%) | 87 (11.03) | 50 (13.59) |
| Hazard ratio (95% CI) | 0.83 (0.58, 1.18) | Reference |
| P value | 0.289 | Reference |
| E value (95% LCI) | 1.70 (1.00) | Reference |
| **All-cause mortality in 5 years** | | |
| No of events (%) | 46 (5.83) | 22 (5.98) |
| Hazard ratio (95% CI) | 0.94 (0.56, 1.57) | Reference |
| P value | 0.802 | Reference |
| E value (95% UCI) | 1.32 (1.00) | Reference |

Patient age, gender, body mass index, systolic blood pressure at admission, and onset date were adjusted. Regarding the outcomes of mRS score, ordinal logistic regression was applied, while binary logistic regression was utilized to examine proportions achieving mRS > 0. For stroke recurrence and all-cause mortality, Cox proportional hazards models were used. CI, confidence interval; LCI, lower confidence interval; mRS, modified Rankin Scale; UCI, upper confidence interval

**Table H. Primary and secondary outcomes of amlodipine users and** **controlled released nifedipine users**

| **Outcome measures** | **Amlodipine (n = 1847)** | **Nifedipine (n = 497)** |
| --- | --- | --- |
| **Primary outcomes** | | |
| **mRS score at 5 years** | | |
| Odds ratio (95% CI) | 0.74 (0.62, 0.90) | Reference |
| P value | 0.002 | Reference |
| E value (95% UCI) | 2.04 (1.46) | Reference |
| **Secondary outcomes** | | |
| **mRS score at 1 year** | | |
| Odds ratio (95% CI) | 0.71 (0.59, 0.86) | Reference |
| P value | <0.001 | Reference |
| E value (95% UCI) | 2.17 (1.60) | Reference |
| **mRS score > 0 at 5 years** |  |  |
| No of events (%) | 956 (51.76) | 298 (59.96) |
| Odds ratio (95% CI) | 0.68 (0.55, 0.84) | Reference |
| P value | <0.001 | Reference |
| E value (95% UCI) | 2.30 (1.67) | Reference |
| **Stroke recurrence in 5 years** | | |
| No of events (%) | 249 (13.48) | 55 (11.07) |
| Hazard ratio (95% CI) | 1.24 (0.92, 1.66) | Reference |
| P value | 0.157 | Reference |
| E value (95% LCI) | 1.79 (1.00) | Reference |
| **All-cause mortality in 5 years** | | |
| No of events (%) | 131 (7.09) | 35 (7.04) |
| Hazard ratio (95% CI) | 0.94 (0.65, 1.37) | Reference |
| P value | 0.764 | Reference |
| E value (95% UCI) | 1.32 (1.00) | Reference |

Patient age, gender, body mass index, systolic blood pressure at admission, and onset date were adjusted. Regarding the outcomes of mRS score, ordinal logistic regression was applied, while binary logistic regression was utilized to examine proportions achieving mRS > 0. For stroke recurrence and all-cause mortality, Cox proportional hazards models were used. CI, confidence interval; LCI, lower confidence interval; mRS, modified Rankin Scale; UCI, upper confidence interval

**Table I. Primary and secondary outcomes of amlodipine users and nifedipine users using Fine-Gray competing risk models**

| **Outcome measures** | **Amlodipine (n = 1847)** | **Nifedipine (n = 1058)** |
| --- | --- | --- |
| **Primary outcomes** | | |
| **mRS score at 5 years** | | |
| Odds ratio (95% CI) | 0.77 (0.67, 0.89) | Reference |
| P value | <0.001 | Reference |
| E value (95% UCI) | 1.92 (1.50) | Reference |
| **Secondary outcomes** | | |
| **mRS score at 1 year** | | |
| Odds ratio (95% CI) | 0.76 (0.66, 0.88) | Reference |
| P value | <0.001 | Reference |
| E value (95% UCI) | 1.96 (1.53) | Reference |
| **mRS score > 0 at 5 years** |  |  |
| No of events (%) | 956 (51.76) | 632 (59.74) |
| Odds ratio (95% CI) | 0.71 (0.61, 0.83) | Reference |
| P value | <0.001 | Reference |
| E value (95% UCI) | 2.17 (1.70) | Reference |
| **Stroke recurrence in 5 years** | | |
| No of events (%) | 249 (13.48) | 147 (13.89) |
| Hazard ratio (95% CI) | 1.00 (0.80, 1.25) | Reference |
| P value | 0.987 | Reference |
| E value (95% LCI) | 1.00 (1.00) | Reference |
| **All-cause mortality in 5 years** | | |
| No of events (%) | 131 (7.09) | 82 (7.75) |
| Hazard ratio (95% CI) | 0.89 (0.67, 1.17) | Reference |
| P value | 0.393 | Reference |
| E value (95% UCI) | 1.50 (1.00) | Reference |

Patient age, gender, body mass index, systolic blood pressure at admission, and onset date were adjusted. Regarding the outcomes of mRS score, ordinal logistic regression was applied, while binary logistic regression was utilized to examine proportions achieving mRS > 0. For stroke recurrence and all-cause mortality, Cox proportional hazards models were used. CI, confidence interval; LCI, lower confidence interval; mRS, modified Rankin Scale; UCI, upper confidence interval

**Table J. Primary and secondary outcomes of amlodipine users and nifedipine users in patients with recurrent stroke**

| **Outcome measures** | **Amlodipine (n = 250)** | **Nifedipine (n = 147)** |
| --- | --- | --- |
| **Primary outcomes** | | |
| **mRS score at 5 years** | | |
| Odds ratio (95% CI) | 0.93 (0.63, 1.36) | Reference |
| P value | 0.691 | Reference |
| E value (95% UCI) | (1.00) | Reference |
| **Secondary outcomes** | | |
| **mRS score at 1 year** | | |
| Odds ratio (95% CI) | 0.90 (0.62, 1.30) | Reference |
| P value | 0.567 | Reference |
| E value (95% UCI) | 1. (1.00) | Reference |
| **mRS score > 0 at 5 years** |  |  |
| No of events (%) | 187 () | 116 () |
| Odds ratio (95% CI) | 0.79 (0.47, 0.31) | Reference |
| P value | 0.354 | Reference |
| E value (95% UCI) | (1.) | Reference |
| **All-cause mortality in 5 years** | | |
| No of events (%) | 40 () | 26 () |
| Hazard ratio (95% CI) | 0.88 (0.53, 1.46) | Reference |
| P value | 0.625 | Reference |
| E value (95% UCI) | 1. (1.00) | Reference |

Patient age, gender, body mass index, systolic blood pressure at admission, and onset date were adjusted. Regarding the outcomes of mRS score, ordinal logistic regression was applied, while binary logistic regression was utilized to examine proportions achieving mRS > 0. For stroke recurrence and all-cause mortality, Cox proportional hazards models were used. CI, confidence interval; LCI, lower confidence interval; mRS, modified Rankin Scale; UCI, upper confidence interval

**Table K. Primary and secondary outcomes of amlodipine users and nifedipine users in patients without recurrent stroke**

| **Outcome measures** | **Amlodipine (n = 1597)** | **Nifedipine (n = 911)** |
| --- | --- | --- |
| **Primary outcomes** | | |
| **mRS score at 5 years** | | |
| Odds ratio (95% CI) | 0.74 (0.63, 0.87) | Reference |
| P value | <0.001 | Reference |
| E value (95% UCI) | (1.00) | Reference |
| **Secondary outcomes** | | |
| **mRS score at 1 year** | | |
| Odds ratio (95% CI) | 0.74 (0.64, 0.87) | Reference |
| P value | <0.001 | Reference |
| E value (95% UCI) | 1. (1.) | Reference |
| **mRS score > 0 at 5 years** |  |  |
| No of events (%) | 91 () | 56 () |
| Odds ratio (95% CI) | 0.69 (0.59, 0.82) | Reference |
| P value | <0.001 | Reference |
| E value (95% UCI) | (1.) | Reference |
| **All-cause mortality in 5 years** | | |
| No of events (%) | 769 () | 516 () |
| Hazard ratio (95% CI) | 0.86 (0.63, 1.24) | Reference |
| P value | 0.473 | Reference |
| E value (95% UCI) | 1. (1.00) | Reference |

Patient age, gender, body mass index, systolic blood pressure at admission, and onset date were adjusted. Regarding the outcomes of mRS score, ordinal logistic regression was applied, while binary logistic regression was utilized to examine proportions achieving mRS > 0. For stroke recurrence and all-cause mortality, Cox proportional hazards models were used. CI, confidence interval; LCI, lower confidence interval; mRS, modified Rankin Scale; UCI, upper confidence interval
